# The effect of bilateral knee osteoarthritis on spatiotemporal gait parameters during incline walking: implications for gait rehabilitation

**DOI:** 10.7717/peerj.20910

**Published:** 2026-03-06

**Authors:** Zhuo Wang, Yue Hou, Lin Yang, Jung H. Chien

**Affiliations:** 1 Rehabilitation Medicine Center and Institute of Rehabilitation Medicine, West China Hospital, Sichuan University, Chengdu, Sichuan, China; 2 Key Laboratory of Rehabilitation Medicine in Sichuan Province, West China Hospital, Sichuan University, Chengdu, Sichuan, China; 3 Life University, Marietta, GA, United States of America

**Keywords:** Bilateral knee osteoarthritis, Incline walking, Gait variability, Rehabilitation, Motor control

## Abstract

**Background:**

Gait alterations in knee osteoarthritis (KOA) patients are well documented; however, there is a paucity of research examining how bilateral KOA patients adapt to incline walking, a common real-world activity. Since KOA patients typically walk slower than healthy adults, it is essential to know if gait differences persist when walking speed is accounted for as a covariate.

**Purpose:**

The aim of this study was to compare spatiotemporal adaptations and gait variability in bilateral KOA patients versus healthy controls over multiple inclines when controlling for walking speed as a covariate.

**Methods:**

Fifteen bilateral KOA patients and fifteen healthy controls were recruited. Gait parameters were collected using a three-dimensional motion analysis system while walking at a self-selected speed on a treadmill at five inclines (+6%, +3%, 0%, −3%, and −6%). A mixed two-way repeated measures Analysis of Covariance (ANCOVA) was used with walking speed as a covariate. Pearson correlations were used to assess the relationship between Western Ontario and McMaster Universities Osteoarthritis Index (WOMAC) score and spatiotemporal parameters.

**Results:**

The ANCOVA revealed a significant group-by-incline interaction for several spatiotemporal parameters. Despite speed being a covariate in the ANCOVA, bilateral KOA patients had significantly shorter step and stride lengths at all inclines and longer double support times during inclined walking (predominantly downhill) compared to the control group. Additionally, KOA patients showed a significant quadratic, U-shaped trend in gait variability such that variability increased during both uphill and downhill walking, while variability linearly decreased in the control group as the incline increased. WOMAC physical function correlated positively with double support time and negatively with step and stride length.

**Conclusion:**

Bilateral KOA patients take a safety-first approach to walking characterized by shorter steps and prolonged double support times to ensure stability and load distribution even at the cost of gait efficiency. The distinct quadratic modulation of gait variability in bilateral KOA patients may represent a novel adaptive motor control strategy for managing the mechanical demands of inclined surfaces. This work could have implications for the rehabilitation of downhill walking in KOA patients.

## Introduction

Knee osteoarthritis (KOA) has recently been identified as a leading cause of global disability. As a result, the prevalence of KOA is a public health concern worldwide. In China, the prevalence rose to nearly 11 million cases in 2021, an increase of over 100% since the early 1990s (Lv et al., 2025). In financial terms, osteoarthritis costs between 1% and 2.5% of global gross domestic product (GDP) and over $140 billion to the United States economy alone (Hawker & King, 2022). The need for rehabilitation, therefore, is pressing. As KOA is a pathology primarily affecting mobility, a characterization of walking alterations is a useful target in order to progress the rehabilitation process.

KOA patients have been consistently shown to walk with a reduced walking speed compared to healthy controls (Suzuki et al., 2023; Kluge et al., 2018). This protective strategy, in conjunction with reduced knee range of motion, has been theorized to be in an attempt to reduce ground reaction forces and joint pain (Suzuki et al., 2023). In this manner, KOA walking is characterized by a spatiotemporal gait signature: short step and stride length, increased double support time, reduced stride frequency, and increased stride time variability (Senden et al., 2011; Bolink et al., 2012; Kluge et al., 2018; Hafer et al., 2020; Odonkor et al., 2020; Ismailidis et al., 2021; Boekesteijn et al., 2022; McCarthy et al., 2013; Rahman et al., 2015). Similar changes have been noted in the healthy elderly, where gait slowing (approx. 12–16% per decade) is largely attributed to a reduction in step length and an increase in double support time (Hollman, McDade & Petersen, 2011). Critically, however, these spatiotemporal differences between older and younger adults are eliminated when both groups are matched for walking speed (Herssens et al., 2020). As KOA patients walk at least 20% slower than age-matched controls (Boekesteijn et al., 2022), the failure to speed-match the data risks attributing this protective slowing strategy directly to pathology. Therefore, in order to provide an accurate analysis of gait in KOA patients, walking speed must be controlled as a covariate.

Gait variability serves as a sensitive sensorimotor marker of the nervous system’s ability to regulate the spatiotemporal characteristics of an inherently rhythmic motor pattern across different environmental constraints (Xie, Liang & Chien, 2023). This parameter typically exhibits a U-shaped relationship with task difficulty, a pattern well-established in the context of walking speed (Jordan, Challis & Newell, 2007; Chien et al., 2015). Generally, step-to-step fluctuations are minimized at a preferred cruising speed and increase markedly at deviations above and below this optimum (Jordan, Challis & Newell, 2007), a phenomenon observed regardless of age (Chien et al., 2015). It is hypothesized that incline angles exert a similar nonlinear constraint on the locomotor system. Xie & Chien (2024) recently provided empirical support for this, demonstrating that step length variability exhibits a significant nonlinear response (quadratic trend, U shape) across different incline conditions. They found that variability was lowest during level walking and increased in both uphill (+8%, +15%) and downhill (−8%, −15%) conditions, suggesting that any deviation from level ground disrupts the habitual locomotor pattern. From this perspective, level walking can be viewed as a condition of high automaticity (low gait variability), driven by spinal and subcortical pathways with minimal higher-level demand (Perrey, 2014; Clark, 2015). In contrast, introducing an incline shifts the locomotor system from automaticity toward executive control. Walking on an incline requires increased supraspinal engagement, specifically of the prefrontal and sensorimotor cortices, to facilitate increased stability demands (Bradford, Lukos & Ferris, 2016). This cortical recruitment is particularly heightened during downhill walking. Mazerie et al. (2012) found that oxyhemoglobin levels in these executive regions were significantly higher during downhill than level or uphill walking, a finding they attributed to the attentional demands of eccentric control. This shift from automaticity to cortical interference subsequently alters the temporal stability of the motor pattern, resulting in increased spatiotemporal variability (Clark, 2015). Based on Stergiou, Harbourne & Cavanaugh (2006) optimal variability hypothesis, this nonlinear fluctuation (quadratic trend) indicates successful adaptation to environmental constraints, as no falls or trips were observed in previous studies. However, it remains unknown whether this modulation of variability is intact in bilateral KOA patients, who possess an intrinsic instability in the knee joint.

In addition to speed considerations, the generally statistically significant negative correlations between WOMAC scores and spatiotemporal gait parameters are generally interpreted as an inverse relationship between patient-reported disability and dynamic motor behavior (Li et al., 2022). In other words, high WOMAC scores (*i.e.,* high pain and stiffness) directly relate to the protective gait pattern, such as reduced self-selected walking speed (Suzuki et al., 2023), which is then directly associated with shorter step lengths (Senden et al., 2011) and reduced stride frequency (Ismailidis et al., 2021). The relationship between WOMAC scores and gait adaptations during level walking is well-established (Li et al., 2022), but the specific relationship between symptom severity and gait performance on an incline is poorly documented. Therefore, it remains unknown whether higher WOMAC scores are directly related to a proportional worsening in gait mechanics as the incline is varied.

While insightful, much of the prior research comparing unilateral and bilateral KOA patients, including that of Messier et al. (2016), suggests two very different forms of coping: inter-limb compensation and systemic adaptation (Creaby, Bennell & Hunt, 2012; Al-Zahrani & Bakheit, 2002). In unilateral KOA patients, the patient is able to lean into asymmetry; as there is an unaffected limb, they are able to offload the painful joint, resulting in a gait pattern defined by that asymmetry (Creaby, Bennell & Hunt, 2012; Al-Zahrani & Bakheit, 2002). When walking on an incline, this asymmetry would be exaggerated as the “good” leg would be responsible for the majority of the propulsive or braking burden. In bilateral KOA patients, there is no longer a healthy compensatory limb. As a result, whole-body, systemic adaptations such as reduced walking speed, uniform step shortening, and increased double support time are made in order to accommodate for the total mechanical load. Therefore, bilateral KOA should not be simply treated as a more severe form of unilateral KOA as this confounds the unique symmetrical strategies that may be adopted. Therefore, this study is limited to the examination of bilateral KOA patients in order to better characterize the systemic locomotor deficits that present when inter-limb compensation is not an option, particularly under the added mechanical constraints of uphill and downhill walking.

Inclined walking has recently been recognized as a particularly rehabilitative exercise for bilateral KOA patients (Sedaghatnezhad et al., 2021). Biomechanically, one of the benefits of walking uphill is an increased glute, hamstring, and quadriceps activation that strengthens these joint stabilizing muscle groups and reduces the risk of injury. Uphill exercise training has also been demonstrated as an effective intervention in non-orthopedic populations. For example, when Chronic Obstructive Pulmonary Disease (COPD) patients paired uphill treadmill training with physical therapy, they saw significantly greater improvements in stride length, walking speed, and knee flexibility than when they just underwent physical therapy (Erfani et al., 2015). On the other hand, in the face of the well-documented uphill walking benefits, downhill walking has not been studied nearly as much. As such, the spatiotemporal gait characteristics and compensatory strategies used by bilateral KOA patients in order to meet the unique mechanical demands of downhill walking are currently unknown.

The present study, therefore, was designed to fill these knowledge gaps by examining the effect of bilateral KOA on spatiotemporal gait parameters during walking on a variety of inclines. This study had two novel design features: (1) controlling for walking speed as a covariate; and (2) the inclusion of downhill walking as a specific condition. We hypothesized that: (1) after controlling for walking speed, bilateral KOA patients would still demonstrate shorter step and stride lengths with longer double support times and wider step widths than healthy controls, regardless of incline; (2) downhill walking would exaggerate these spatiotemporal deviations to a significantly greater extent than level or uphill walking; and (3) a negative correlation between WOMAC scores and step/stride length and a positive correlation between WOMAC scores and double support time would be found, regardless of the walking incline.

## Materials & Methods

### Participants

This study received approval from the Institutional Review Board of West China Hospital, Sichuan University #2024-1637. Written informed consent was obtained from all participants, including both healthy controls and bilateral KOA patients. In total, 15 bilateral KOA patients (eight females) and 15 healthy controls (eight females), matched for age, height, weight, and walking speed, were enrolled through West China Hospital. The mean age (years) was 61.60 ± 8.69 for the bilateral KOA patients and 55.13 ± 10.74 for the control group; mean height (cm) was 162.33 ± 9.55 for the bilateral KOA patients and 161.13 ± 7.51 for the controls; mean weight (kg) was 64.17 ± 11.96 for the bilateral KOA patients and 61.70 ± 10.44 for the controls; and mean walking speed (km/hr) was 2.53 ± 0.79 for the bilateral KOA patients and 2.74 ± 0.72 for the controls. More details are provided in Table 1.

**Table 1:** Participants’ information. Unit: Age: years old, Height/ Leg length: cm, Weight: kg, KOA: bilateral KOA patients.

	Gender	Age	Height (leg length)	Weight	WOMAC -Pain	WOMAC -Stiffness	WOMAC -Physical function	WOMAC overall	WS (km/h)		Gender	Age	Height (leg length)		weight	WS (km/h)
KOA01	Female	42	158 (79)	50	8	2	14	24	1.9	Con01	Female	53	156 (78)		50	2
KOA02	Male	69	176 (71)	72	2	2	7	11	3.8	Con02	Male	33	175 (71)		70	3.7
KOA03	Female	61	150 (75)	59	3	1	26	30	1.9	Con03	Female	55	159 (75)		57	1.9
KOA04	Male	66	170 (71)	68	5	2	10	17	2	Con04	Male	37	170 (71)		65	2.7
KOA05	Female	65	158 (79)	57	4	0	13	17	2.4	Con05	Female	50	163 (82)		53	2.1
KOA06	Male	64	175 (83)	85	8	3	22	33	2	Con06	Male	57	164 (82)		80	2.5
KOA07	Male	54	166 (83)	78	8	0	28	36	2	Con07	Male	70	168 (81)		70	2
KOA08	Female	55	168 (84)	57	3	1	26	30	2	Con08	Female	66	150 (75)		63	2.1
KOA09	Female	73	150 (75)	59	2	0	18	20	2.1	Con09	Female	50	152 (76)		52	3.3
KOA10	Male	72	155 (75)	65	8	5	31	44	1.9	Con10	Male	62	164 (82)		65	2.8
KOA11	Male	72	170 (85)	86	2	1	10	13	2.4	Con11	Male	60	160 (80)		79	3.1
KOA12	Female	61	152 (76)	52	10	2	30	42	2.3	Con12	Female	73	163 (85)		60	2.1
KOA13	Female	63	162 (81)	55	6	0	17	23	4	Con13	Female	53	164 (82)		65	3.7
KOA14	Male	54	174 (82)	70	5	2	17	24	3.3	Con14	Male	55	162 (81)		53	4
KOA15	Female	53	151 (75)	50	2	2	13	17	3.9	Con15	Female	53	147 (75)		44	3.2
	8F/7M	61.6	162.33 (78)	64.17	5.06	1.53	18.8	25.4	2.53		8F/7M	55.13		161.13 (78)	61.7	2.74

**Notes.**

WS walking speed

According to a cross-sectional comparison of three sets of clinical classification criteria, the National Institute for Health and Care Excellence (NICE) can identify most KOA patients (Skou et al., 2020). Thus, in this study, patients can be diagnosed with knee OA if they have activity-related joint pain and have either no morning knee stiffness or stiffness that lasts no longer than 30 min. However, while NICE guidelines prioritize a clinical-first approach to ensure high sensitivity in diagnosis, this study also incorporates the Osteoarthritis Research Society International (OARSI) recommendation for objective physical tests. To ensure a homogenous sample for biomechanical analysis, these clinical frameworks were further constrained by specific structural and functional thresholds. Radiographic precision was maintained using the Kellgren-Lawrence (K/L) scale, specifically targeting Grades 2 and 3 (mild to moderate) to verify structural pathology. Additionally, a WOMAC score of ≤ 45 was utilized as an inclusion criterion. As suggested by Riddle & Stratford (2013), scores in the 25–45 range represent a moderate impact on quality of life, which is the ideal population for testing rehabilitative exercises like incline walking. This specific upper limit also served as a safety threshold, ensuring that participants possessed sufficient physical capacity to navigate the challenging −6% downhill grade without the confounding effects of severe end-stage disability. By combining the clinical reach of NICE and OARSI with explicit K/L and WOMAC cutoffs, this study isolates the stability-first motor control signatures unique to bilateral KOA patients. Exclusion criteria for both bilateral KOA patients and controls were as follows (Haggerty et al., 2014): (1) mental or psychiatric disorders affecting gait; (2) cardiovascular, pulmonary, or cerebral conditions impacting gait; (3) a history of surgery involving the lower extremities or spine; (4) a diagnosis of rheumatoid arthritis; and (5) intraarticular corticosteroid injection within the preceding two months. Controls were matched as closely as possible to the bilateral KOA patients based on baseline characteristics (height, weight, and walking speed). Before data collection, all participants were informed that they would be required to walk on a treadmill at varying inclines.

The sample size calculation was based on our preliminary results. We firstly recruited 20 participants (10 bilateral KOA patients and 10 healthy individuals) and analyzed double support time, as this parameter has been shown to be critical for identifying gait performance differences between bilateral KOA patients and healthy individuals (Zhang et al., 2024). A two-way mixed repeated measures ANOVA revealed a significant interaction between incline and group on double support time (*p* < 0.001), with a Partial Eta Squared (${\eta }_{\mathbi{p}}^{2}$ ) of 0.466.

\begin{eqnarray*}\mathbi{f}=\sqrt{ \frac{{\eta }_{\mathbi{p}}^{2}}{1-{\eta }_{\mathbi{p}}^{2}} }. \end{eqnarray*}

This ${\eta }_{\mathbi{p}}^{2}$ value was converted to Cohen’s *f* (*f* = 0.93) to estimate the required sample size using G*Power (Faul et al., 2007). The analysis indicated that a total sample size of 27 (approximately 13 per group) is required to achieve 95% power. This approach aligns with similar research in the field, where smaller sample sizes are justified when large effect sizes are anticipated and the primary outcome involves gait symmetry or related biomechanical parameters (Haggerty et al., 2014; Higgins et al., 2024; Wang, Chien & He, 2025).

### Experimental design and protocol

Gait events, such as heel strikes and toe-offs, were captured using reflective markers at 120 Hz with a motion capture system consisting of eight cameras (Arqus A9, 3D resolution: 0.05 mm, Qualisys AB, Gothenburg, Sweden) at West China Hospital. Retroreflective markers were attached to the seventh cervical vertebra (C7), tenth thoracic vertebra (T10), jugular notch, xiphoid process, anterior superior iliac spine, posterior superior iliac spine, greater trochanter, medial femoral epicondyle, lateral femoral epicondyle, thigh, tibia, medial malleolus, lateral malleolus, posterior calcaneus, medial calcaneus, lateral calcaneus, heel, and the 2nd (M2) and 5th (M5) metatarsal heads based on Plug in Gait model (Josep & Darras, 2023). The marker sizes were four mm. However, for the calculation of spatiotemporal gait parameters in the current study-such as double support time, stance time, step length, stride length, step width, step time, and stride time, only two markers, the toe (M2) and heel, were used on each leg. The markers were designed with maximum roundness to ensure effective capture of foot movement. After calibration, the average residual value from each camera had to be below 0.4 mm to ensure data accuracy; otherwise, recalibration was required. A treadmill equipped with a safety lanyard (X24 Treadmill, NTL39225, NordicTrack, Logan, UT, USA) was used for both patients and controls to perform walking at various inclines (−6%, −3%, 0%, 3%, 6%). The treadmill belt area was 55 × 152 cm, the incline range was −6% to 40%, the maximum user capacity was 182 kg, and the speed limit was 19 km/h. It is worth mentioning that toe and heel marker trajectories were filtered using a low-pass Butterworth digital filter with a cut-off frequency of six Hz (Rácz & Kiss, 2021).

**Figure 1:**
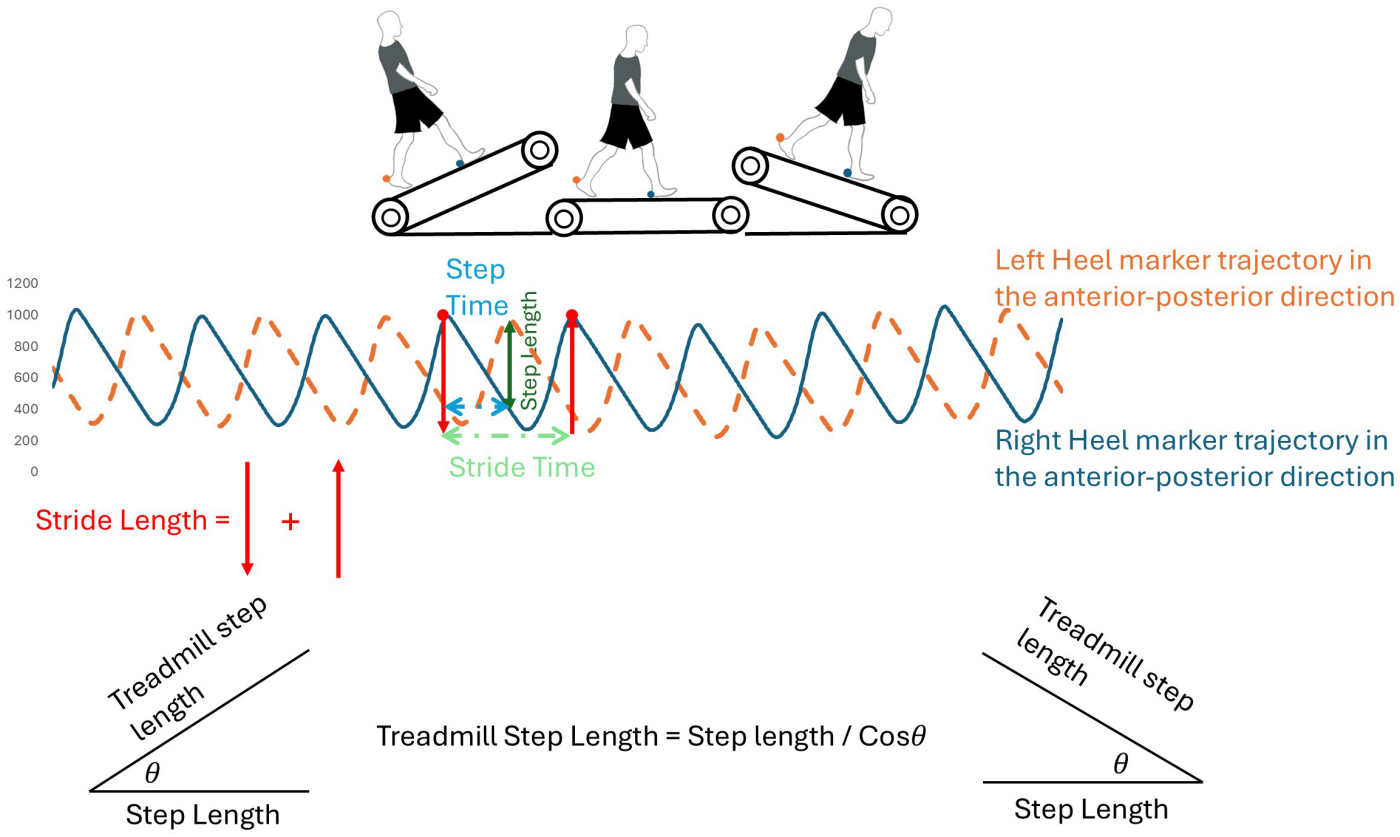
Illustration of gait cycle events and spatiotemporal definitions. This figure defines the primary gait parameters used in the study. Step length and step time are derived from the interval between a heel strike on one leg and the subsequent heel strike of the opposite leg. Stride length and stride time represent the duration and distance between two consecutive heel strikes of the same leg.

A gait cycle is defined as the duration between two consecutive heel strikes of the same leg, identified when horizontal heel displacement reaches its maximum, determined by heel marker trajectories in the anterior-posterior direction (Xie, Liang & Chien, 2023, Fig. 1). Step time refers to the interval between a heel strike on one leg and the subsequent heel strike of the opposite leg (Fig. 1). Step length is defined as the straight-line distance in the sagittal plane between consecutive heel strikes (Fig. 1). Because using only horizontal distance between markers inaccurately represents step length on inclines, step and stride lengths were adjusted using the cosine of the incline angle. Therefore, step and stride lengths during inclined walking were calculated using the formula: measured length divided by the cosine of ±1.72° for a  ± 3% grade or divided by the cosine of  ± 3.43° for a  ± 6% grade. Step width is defined as the lateral distance between heel markers from one heel strike to the subsequent contralateral heel strike. Stance time is defined as the duration between a heel strike event and toe-off event in the same leg. Double support time is the duration both feet are in contact with the treadmill belt during walking. To ensure methodological consistency across all groups and conditions, mean and variability (Coefficient of Variation) values were calculated using a standardized window of the first 40 gait cycles (80 steps) for all participants. This specific duration was selected to match the maximum data collected from a single bilateral KOA patient participant during the 6% incline condition, thereby eliminating potential biases associated with varying data lengths. The dependent variables were double support time, stance time, step/stride length, step width, step/stride time and respective variabilities.

For experimental protocol, the first step was to determine participants’ preferred walking speed (PWS), they first stood on the side of treadmill while an experimenter set the speed to one km/h. After walking for 30 s, participants checked if this speed felt comfortable, like walking on a typical street. The speed was then adjusted by increments of 0.1 km/h until participants confirmed their PWS. Participants tried out five different inclines to choose their final PWS. After resting for two minutes, they completed five random walking trials on different inclines (+6%, +3%, 0%, −3%, and −6%). The order of these trials was randomized for each subject using the RAND function in Microsoft Excel to minimize order effects. This study chose to control walking speed (participants kept the same his/her PWS across all inclines) across all inclines for two primary reasons: isolation of independent variables and safety and comfort verification. By keeping speed constant, this study ensured that any observed differences in gait mechanics could be attributed solely to the change in incline (the mechanical constraint) rather than a secondary alteration in walking velocity. Also, participants tested the speed across the five different inclines prior to data collection to ensure the chosen velocity was comfortable and safe for both the steepest uphill (+6%) and downhill (−6%) conditions. Each trial lasted two minutes, with a mandatory two-minute rest between each one, as recommended by Kempski et al.’s (2018) study. More rest time was provided if needed.

### Statistical analysis

Normality of dependent variables was assessed using the Shapiro–Wilk test. For normally distributed data, a mixed two-way repeated measures ANCOVA (with walking speed as a covariate) was used to assess the interaction between locomotor tasks (incline) and group (bilateral KOA patients *vs.* Controls) on spatiotemporal parameters. Also, the test of sphericity (most commonly Mauchly’s Test) is a critical procedure used when performing a Repeated Measures ANOVA. It determines whether the variances of the differences between all possible pairs of within-subject conditions are equal. If sphericity was met (*p* > 0.05), the standard “Sphericity Assumed” F-test was used. In contrast, if sphericity was violated, correction (Huynh-Feldt) was used to adjust the degree of freedom. If a significant interaction was found, pairwise comparisons with Bonferroni corrections were performed. For non-normally distributed data, a non-parametric longitudinal data model (Brunner, Domhof & Langer, 2002) was utilized to investigate within-subject (five different inclines) and between-subject (bilateral KOA patients and Controls) effects. *Post-hoc* pairwise comparisons were conducted using Wilcoxon signed-rank tests (within-group) and Mann–Whitney U tests (between-group). To control for the error rate across multiple comparisons, all *p*-values were adjusted using the Holm–Bonferroni method. Statistical significance was defined as an adjusted *p* < 0.05.

To evaluate the trends between inclination angle and gait parameters, Linear Mixed Models (LMM) were employed. If a significant group-by-incline interaction was detected (*p* < 0.05), separate LMMs were applied for each group to characterize the trends across the incline conditions (−6% to +6%). Pearson correlations were calculated to evaluate relationships between spatiotemporal variables across different inclines as well as WOMAC scores (pain, stiffness, and physical function). All statistical analyses were performed using SPSS version 31 (IBM, Armonk, NY, USA).

## Results

### The normality of dependent variables

The *p*-values of the Shapiro–Wilk test were greater than 0.05 for step/stride length, step/stride time, double support time, stance time, step width, and their respective variabilities, indicating that the data were normally distributed. Therefore, a mixed two-way repeated measures ANCOVA was used to analyze these spatiotemporal parameters.

### The interaction of each dependent variable between bilateral KOA patients and healthy controls

Mauchly’s test of Sphericity revealed that *p* value was smaller than 0.05 for most of spatiotemporal parameters except step width, stride length variability, step/stride time variability. Therefore, Huynh-Feldt corrections were used. A significant Group × Incline interaction was found for double support time (*F* = 18.87, *p* < 0.001), double support time variability (*F* = 13.79, *p* < 0.001), stance time (*F* = 9.37, *p* < 0.001), step length (*F* = 7.01, *p* < 0.001), step width (*F* = 6.56, *p* < 0.001), step length variability (*F* = 16.45, *p* < 0.001), stride length (*F* = 7.53, *p* = 0.01), stride length variability (*F* = 11.98, *p* < 0.001), step time variability (*F* = 13.56, *p* < 0.001), and stride time variability (*F* = 9.51, *p* < 0.001). No significant effects of walking speed were observed in any of the statistical analyses.

**Figure 2:**
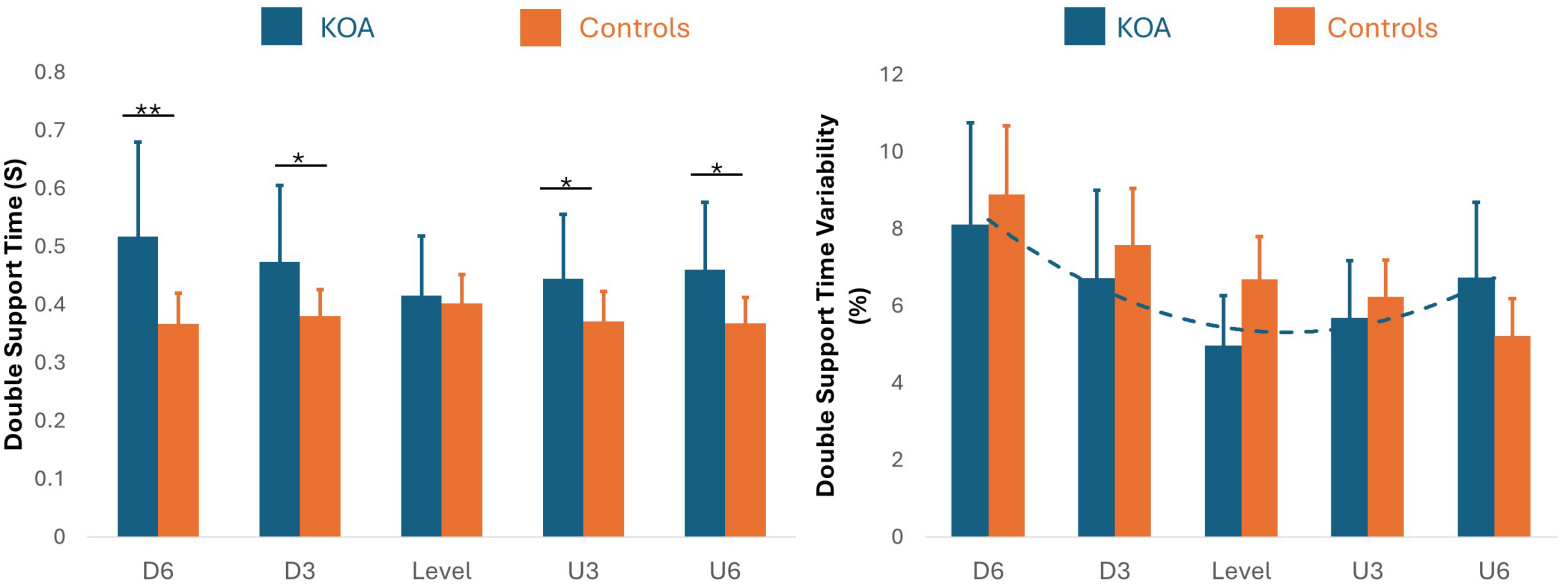
Double support time and double support time variability across inclines. This figure illustrates the temporal adaptations and stability strategies used by patients with bilateral knee osteoarthritis (KOA) compared to healthy controls. Left (Double Support Time): Bilateral KOA patients (blue) demonstrated significantly longer double support times compared to controls (orange) during downhill walking at a −6% grade (*p* < 0.01) and a −3% grade (*p* < 0.05), as well as during uphill walking at +3% and +6% grades (*p* < 0.05). No significant differences were observed between groups during level walking when speed was controlled as a covariate. Right (Double Support Time Variability): The bilateral KOA patients exhibited a significant quadratic, U-shaped trend in gait variability, with increased variability during both uphill and downhill extremes compared to level walking. In contrast, the control group followed a linear decrease in variability as the incline increased. *: *p* < 0.05, **: *p* < 0.01.

**Figure 3:**
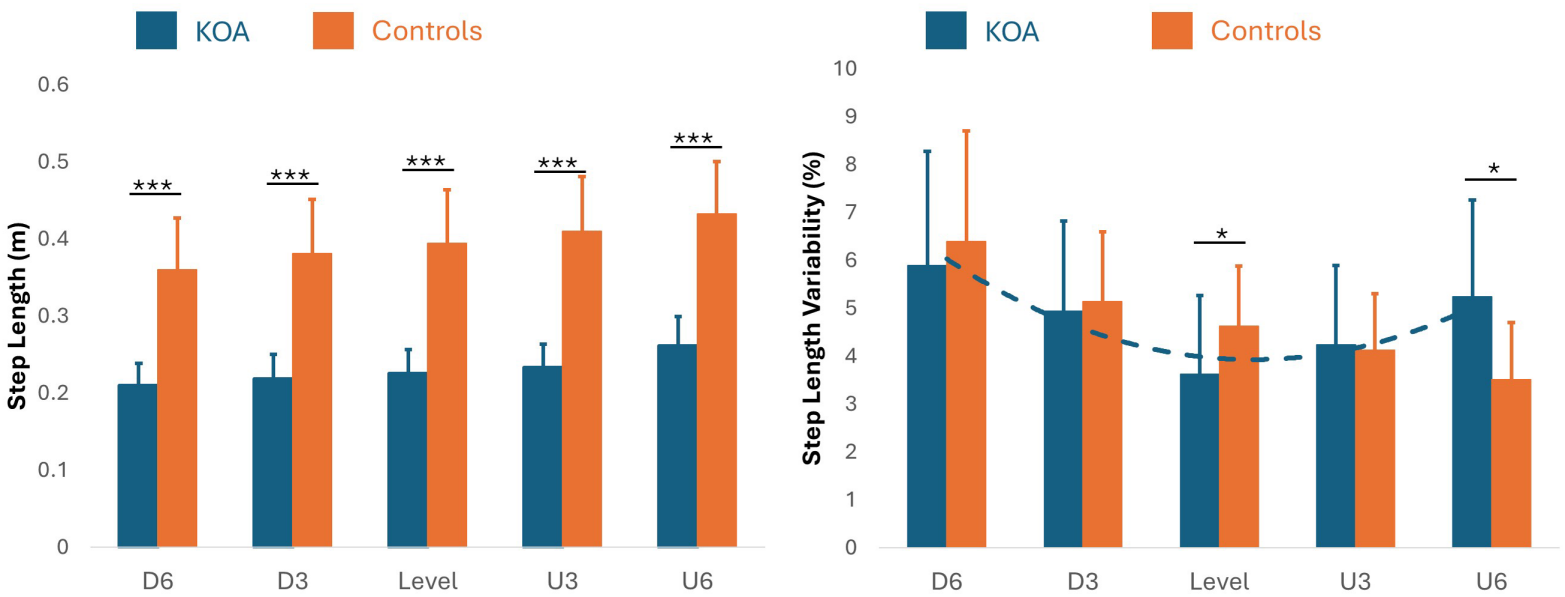
Comparison of double support time and its variability between bilateral KOA patients and controls. This figure illustrates the temporal adaptations and stability strategies used by patients with bilateral KOA patients compared to healthy controls across five inclines (−6%, −3%, 0%, 3%, 6%). Double Support Time (s): Bilateral KOA patients demonstrated significantly longer double support times compared to controls during inclined walking, specifically at −6%, −3%, +3%, and +6% grades. No significant difference was observed during level walking (0%) when speed was controlled as a covariate. Double Support Time Variability (%): The bilateral KOA patients exhibited a significant quadratic, U-shaped trend, with increased variability during both uphill and downhill extremes. In contrast, the control group showed a linear decrease in variability as the incline increased. Statistical Significance: Asterisks denote significant between-group differences (*: *p* < 0.05, ***: *p* < 0.01).

Bonferroni-corrected pairwise comparisons indicated that step and stride lengths were significantly shorter in bilateral KOA patients than in healthy controls across all incline conditions (Table 2, Figs. 2–3). Both groups increased their step and stride lengths as the incline increased from −6% to +6%. Similarly, step and stride times increased for both groups as the incline increased. Regarding double support time, bilateral KOA patients demonstrated significantly longer times when walking on −6%, −3%, +3%, and +6% grades compared to level walking. Specifically, the +6% grade resulted in longer double support times for bilateral KOA patients compared to all other grades. In contrast, controls showed no significant differences in double support time across inclines relative to the +6% condition. Detailed statistics are provided in Tables 2 and 3.

**Table 2:** The statistical analysis of each dependent variable. DST, double support time; ST, stance time; StepL, step length; StrideL, stride length); StepWidth, step width; StrideT, stride time; StepT, step time; IN, effect of incline; IN X WS, the interaction between inclines and walking speed; WS, the effect of walking speed; GR, the effect of different groups; K, bilateral KOA patients; C, healthy controls; PES, partial eta square; (L, U), lower and upper boundary of 95% Confidence Interval for Difference (K–C). *p* = ∗∗(<0.01), *p* = ∗∗∗(<0.001). D, downhill walking; U, uphill walking; 3, or 6, grade of incline.

DST										
IN	*F* = 2.44, *p* = 0.10, PES = 0.083	IN X WS	*F*=0.81, *p* = 0.45, PES = 0.029	IN X GR	*F* = 18.87, *p* = ∗∗∗, PTS = 0.409	WS	*F* = 3.93, *p* = 0.06, PTS = 0.127	GR	F = 5.41, *p* = 0.03, PTS = 0.167	
	D6		D3		Level		U3		U6	
	MEAN	STD	MEAN	STD	MEAN	STD	MEAN	STD	MEAN	STD
K	0.52	0.16	0.47	0.13	0.41	0.11	0.44	0.11	0.46	0.12
C	0.37	0.05	0.38	0.04	0.4	0.05	0.37	0.05	0.37	0.04
K *vs.* C	D6	(L,U)	D3	(L,U)	Level	(L,U)	U3	(L,U)	U6	(L,U)
	*p* = ∗∗	(0.05, 0.23)	*p* = 0.02	(0.01, 0.15)	*p* = 0.84	(−0.05, 0.06)	*p* = 0.04	(0.003, 0.13)	*p* = 0.01	(0.02, 0.15)
	D6 *vs.* D3	D6 *vs.* Level	D6 *vs.* U3	D6 *vs.* U6	D3 *vs.* Level	D3. *vs.* U3	D3 *vs.* U6	Level *vs.* U3	Level *vs.* U6	U3 *vs.* U6
K	*p* = ∗∗∗	*p* = ∗∗∗	*p* = ∗∗	*p* = ∗∗	*p* = ∗∗∗	*p* = 0.06	*p* = 0.53	*p* = 0.01	*p* = ∗∗∗	*p* = 0.27
C	*p* = ∼ 1	*p* = 0.60	*p* = ∼ 1	*p* = ∼ 1	*p* = 0.49	*p* = ∼ 1	*p* = 0.50	*p* = 0.60	*p* = 0.60	*p* = ∼ 1
ST										
IN	*F*=0.74, *p* = 0.49, PES = 0.027	IN X WS	*F*=0.53, *p* = 0.62, PES = 0.019	IN X GR	*F* = 9.37, *p* = ∗∗∗, PES = 0.258	WS	*F* = 3.74, *p* = 0.06, PES = 0.122	GR	F = 2.18, *p* = 0.15, PES = 0.075	
	D6		D3		Level		U3		U6	
	MEAN	STD	MEAN	STD	MEAN	STD	MEAN	STD	MEAN	STD
K	0.91	0.23	0.86	0.13	0.81	0.13	0.85	0.15	0.88	0.18
C	0.75	0.09	0.76	0.09	0.82	0.11	0.79	0.1	0.77	0.09
K *vs.* C	D6	(L,U)	D3	(L,U)	Level	(L,U)	U3	(L,U)	U6	(L,U)
	*p* = 0.04	(0.01, 0.27)	*p* = 0.14	(−0.03, 0.19)	*p* = 0.73	(−0.11, 0.08)	*p* = 0.21	(−0.04, 0.15)	*p* = 0.07	(−0.07, 0.20)
	D6 *vs.* D3	D6 *vs.* Level	D6 *vs.* U3	D6 *vs.* U6	D3 *vs.* Level	D3. *vs.* U3	D3 *vs.* U6	Level *vs.* U3	Level *vs.* U6	U3 *vs.* U6
KOA	*p* = ∗∗	*p* = 0.01	*p* = 0.38	*p* = ∼ 1	*p* = 0.33	*p* = ∼ 1	*p* = ∼ 1	*p* = ∗∗∗	*p* = 0.01	*p* = 0.913
Control	*p* = ∼ 1	*p* = 0.27	*p* = ∼ 1	*p* = ∼ 1	*p* = 0.10	*p* = ∼ 1	*p* = ∼ 1	*p* = 0.04	*p* = 0.17	*p* = ∼ 1
StepL										
IN	*F* = 19.36, *p* = ∗∗∗, PES = 0.418	IN X WS	*F* = 1.81, *p* = 0.15, PES = 0.063	IN X GR	*F* = 7.01, *p* = ∗∗∗, PES = 0.206	WS	*F* = 2.36, *p* = 0.14, PES = 0.08	GR	F=71.67, *p* = ∗∗∗, PES = 0.726	
	D6		D3		Level		U3		U6	
	MEAN	STD	MEAN	STD	MEAN	STD	MEAN	STD	MEAN	STD
K	0.21	0.03	0.22	0.03	0.23	0.03	0.23	0.03	0.26	0.04
C	0.36	0.07	0.38	0.07	0.39	0.06	0.41	0.07	0.43	0.07
K *vs.* C	D6	(L,U)	D3	(L,U)	Level	(L,U)	U3	(L,U)	U6	(L,U)
	*p* = ∗∗∗	(−0.183, −0.108)	*p* = ∗∗∗	(−0.197, −0.118)	*p* = ∗∗∗	(−0.204, −0.124)	*p* = ∗∗∗	(−213, −0.132)	*p* = ∗∗∗	(−0.21, −0.127)
	D6 *vs.* D3	D6 *vs.* Level	D6 *vs.* U3	D6 *vs.* U6	D3 *vs.* Level	D3. *vs.* U3	D3 *vs.* U6	Level *vs.* U3	Level *vs.* U6	U3 *vs.* U6
K	*p* = 0.05	*p* = ∗∗	*p* = ∗∗	*p* = ∗∗∗	*p* = 0.3	*p* = 0.04	*p* = ∗∗∗	*p* = 0.21	*p* = ∗∗∗	*p* = ∗∗∗
C	*p* = ∗∗∗	*p* = ∗∗∗	*p* = ∗∗∗	*p* = ∗∗∗	*p* = ∗∗∗	*p* = ∗∗∗	*p* = ∗∗∗	*p* = ∗∗∗	*p* = ∗∗∗	*p* = ∗∗∗
StrideL										
IN	*F* = 28.44, *p* = ∗∗∗, PES = 0.513	IN X WS	*F* = 2.92, *p* = 0.10, PES = 0.098	IN X GR	*F* = 7.53, *p* = 0.01, PES = 0.218	WS	*F*=0.39, *p* = 0.53, PES = 0.015	GR	F=22.12, *p* = ∗∗, PES = 0.45	
	D6		D3		Level		U3		U6	
	MEAN	STD	MEAN	STD	MEAN	STD	MEAN	STD	MEAN	STD
K	0.53	0.13	0.54	0.13	0.57	0.15	0.59	0.15	0.64	0.16
C	0.77	0.15	0.81	0.16	0.85	0.16	0.88	0.16	0.94	0.17
K *vs.* C	D6	(L,U)	D3	(L,U)	Level	(L,U)	U3	(L,U)	U6	(L,U)
	*p* = ∗∗∗	(−0.34, −0.123)	*p* = ∗∗∗	(−0.368, −0.143)	*p* = ∗∗∗	(−0.388, −0.152)	*p* = ∗∗∗	(−0.405, −0.162)	*p* = ∗∗∗	(−0.415, −0.160)
	D6 *vs.* D3	D6 *vs.* Level	D6 *vs.* U3	D6 *vs.* U6	D3 *vs.* Level	D3. *vs.* U3	D3 *vs.* U6	Level *vs.* U3	Level *vs.* U6	U3 *vs.* U6
K	*p* = 0.03	*p* = ∗∗	*p* = ∗∗	*p* = ∗∗∗	*p* = 0.19	*p* = ∗∗	*p* = ∗∗∗	*p* = 0.11	*p* = ∗∗∗	*p* = ∗∗∗
C	*p* = ∗∗∗	*p* = ∗∗∗	*p* = ∗∗∗	*p* = ∗∗∗	*p* = ∗∗	*p* = ∗∗∗	*p* = ∗∗∗	*p* = ∗∗	*p* = ∗∗∗	*p* = ∗∗∗
StepW										
IN	*F* = 1.02, *p* = 0.39, PES = 0.037	IN X WS	*F*=0.49, *p* = 0.72, PES = 0.018	IN X GR	*F* = 6.56, *p* = ∗∗∗, PES = 0.195	WS	*F* = 1.74, *p* = 0.19, PES = 0.061	GR	F = 0.26, *p* = 0.61, PES = 0.01	
	D6		D3		Level		U3		U6	
	MEAN	STD	MEAN	STD	MEAN	STD	MEAN	STD	MEAN	STD
K	0.12	0.01	0.11	0.01	0.1	0.01	0.11	0.01	0.12	0.01
C	0.11	0.03	0.11	0.03	0.11	0.03	0.1	0.03	0.1	0.03
K *vs.* C	D6	(L,U)	D3	(L,U)	Level	(L,U)	U3	(L,U)	U6	(L,U)
	*p* = 0.08	(−0.002, 0.029)	*p* = 0.82	(−0.016, 0.02)	*p* = 0.50	(−0.024, 0.012)	*p* = 0.64	(−0.014, 0.023)	*p* = 0.45	(−0.012, 0.026)
	D6 *vs.* D3	D6 *vs.* Level	D6 *vs.* U3	D6 *vs.* U6	D3 *vs.* Level	D3. *vs.* U3	D3 *vs.* U6	Level *vs.* U3	Level *vs.* U6	U3 *vs.* U6
K	*p* = 0.02	*p* = ∗∗∗	*p* = ∗∗	*p* = 0.03	*p* = ∗∗	*p* = ∼ 1	*p* = ∼ 1	*p* = 0.21	*p* = 0.04	*p* = ∼ 1
C	*p* = ∼ 1	*p* = ∼ 1	*p* = ∼ 1	*p* = ∼ 1	*p* = ∼ 1	*p* = 0.82	*p* = 0.64	*p* = ∼ 1	*p* = ∼ 1	*p* = ∼ 1
StepT										
IN	*F* = 10.69, *p* = ∗∗∗, PES = 0.284	IN X WS	F 2.74, *p* = 0.08, PES = 0.092	IN X GR	*F*=0.66, *p* = 0.51, PES = 0.024	WS	*F*=0.001, *p* = ∼ 1, PES = ∼0	GR	F = 2.28, *p* = 0.14, PES = 0.142	
	D6		D3		Level		U3		U6	
	MEAN	STD	MEAN	STD	MEAN	STD	MEAN	STD	MEAN	STD
K	0.59	0.09	0.61	0.09	0.63	0.12	0.64	0.11	0.67	0.13
C	0.55	0.05	0.56	0.05	0.57	0.06	0.59	0.07	0.61	0.07
StrideT										
IN	*F* = 11.23, *p* = ∗∗∗, PES = 0.294	IN X WS	*F* = 3.18, *p* = 0.06, PES = 0.105	IN X GR	*F*=0.44, *p* = 0.65, PES = 0.016	WS	*F* = 3.48, *p* = 0.07, PES = 0.114	GR	F = 1.76, *p* = 0.19, PES = 0.061	
	D6		D3		Level		U3		U6	
	MEAN	STD	MEAN	STD	MEAN	STD	MEAN	STD	MEAN	STD
K	1.18	0.19	1.21	0.18	1.26	0.22	1.3	0.24	1.34	0.27
C	1.11	0.11	1.12	0.11	1.15	0.12	1.19	0.15	1.22	0.13

**Table 3:** The statistical analysis of each dependent variable. DSTV (double support time variability), STV (stance time variability), StepLV (step length variability), StrideLV (stride length variability), StepWidthV (step width variability), StrideTV (stride time variability), and StepTV (step time variability). IN: effect of incline, IN X WS: the interaction between inclines and walking speed, WS: the effect of walking speed, GR: the effect of different groups, K: bilateral KOA patients, C: healthy controls, PES: partial eta square, (L, U): lower and upper boundary of 95% Confidence Interval for Difference (K - C). p = ** (<0.01), *p* = ∗∗∗ (<0.001). D: downhill walking, U: uphill walking. 3, or 6: grade of incline.

DSTV																			
IN		F = 6.26, *p* = ∗∗∗, PES = 0.188		IN X WS		F = 0.59, *p* = 0.63, PES = 0.021		IN X GR		F = 13.79, *p* = ∗∗∗, PES = 0.338		WS		F = 3.57, *p* = 0.07, PES = 0.117		GR		F = 1.43, *p* = 0.24, PES = 0.05	
	D6				D3				Level				U3				U6		
	MEAN		STD		MEAN		STD		MEAN		STD		MEAN		STD		MEAN		STD
KOA	8.1		2.64		6.7		2.29		4.96		1.3		5.68		1.49		6.72		1.96
Control	8.88		1.79		7.57		1.47		6.68		1.11		6.23		0.95		5.21		0.97
K *vs.* C	D6		(L,U)		D3		(L,U)		Level		(L,U)		U3		(L,U)		U6		(L,U)
	*p* = 0.23		(−2.65, 0.67)		*p* = 0.17		(−2.45, 0.45)		*p* = ∗∗∗		(−2.72, −0.95)		*p* = 0.12		(−1.59, 0.19)		*p* = 0.02		(0.23, 2.46)
	D6 *vs.* D3		D6 *vs.* Level		D6 *vs.* U3		D6 *vs.* U6		D3 *vs.* Level		D3. *vs.* U3		D3 *vs.* U6		Level *vs.* U3		Level *vs.* U6		U3 *vs.* U6
K	*p* = ∗∗		*p* = ∗∗∗		*p* = ∗∗∗		*p* = 0.03		*p* = ∗∗∗		*p* = ∗∗		*p* = ∼ 1		*p* = ∗∗		*p* = ∗∗∗		*p* = ∗∗
C	*p* = ∗∗		*p* = ∗∗∗		*p* = ∗∗∗		*p* = ∗∗∗		*p* = 0.06		*p* = ∗∗∗		*p* = ∗∗∗		*p* = 0.17		*p* = ∗∗∗		*p* = ∗∗
STV																			
IN		F = 1.45, *p* = 0.22, PES = 0.051		IN X WS		F = 0.89, *p* = 0.46, PES = 0.032		IN X GR		F = 0.35, *p* = 0.82, PES = 0.013		WS		F = 2.39, *p* = 0.13, PES = 0.082		GR		F = 1.68, *p* = 0.21, PES = 0.058	
	D6				D3				Level				U3				U6		
	MEAN		STD		MEAN		STD		MEAN		STD		MEAN		STD		MEAN		STD
K	4.33		1.35		4.32		1.35		4.86		2.09		4.42		1.71		4.06		0.83
C	3.58		0.85		3.87		1.21		4.09		0.92		4.11		1.44		3.28		1.3
StepLV																			
IN		F = 7.71, *p* = ∗∗∗, PES = 0.222		IN X WS		F = 1.42, *p* = 0.25, PES = 0.05		IN X GR		F = 16.45, *p* = ∗∗∗, PES = 0.379		WS		F = 1.72, *p* = 0.2, PES = 0.06		GR		F = 0.02, *p* = 88, PES = 0.001	
	D6				D3				Level				U3				U6		
	MEAN		STD		MEAN		STD		MEAN		STD		MEAN		STD		MEAN		STD
K	5.89		2.38		4.94		1.88		3.63		1.63		4.23		1.66		5.24		2.01
C	6.39		2.31		5.14		1.45		4.63		1.25		4.12		1.18		3.51		1.19
K *vs.* C	D6		(L,U)		D3		(L,U)		Level		(L,U)		U3		(L,U)		U6		(L,U)
	p = 0.42		(−2.42, 1.04)		*p* = 0.65		(−1.57, 0.99)		*p* = 0.06		(−2.19, −0.02)		*p* = ∼ 1		(−1.07, 1.08)		*p* = 0.01		(0.39, 2.89)
	D6 *vs.* D3		D6 *vs.* Level		D6 *vs.* U3		D6 *vs.* U6		D3 *vs.* Level		D3. *vs.* U3		D3 *vs.* U6		Level *vs.* U3		Level *vs.* U6		U3 *vs.* U6
K	*p* = 0.02		*p* = ∗∗∗		*p* = ∗∗		*p* = 0.87		*p* = ∗∗∗		*p* = ∗∗		*p* = ∼ 1		*p* = ∗∗∗		*p* = ∗∗∗		*p* = ∗∗∗
C	*p* = ∗∗∗		*p* = ∗∗∗		*p* = ∗∗∗		*p* = ∗∗∗		*p* = 0.22		*p* = ∗∗∗		*p* = ∗∗∗		*p* = ∗∗		*p* = ∗∗∗		*p* = 0.01
StrideLV																			
IN		F = 2.13, *p* = 0.08, PES = 0.073		IN X WS		F = 0.16, *p* = 0.96, PES = 0.006		IN X GR		F = 11.98, *p* = ∗∗∗, PES = 0.307		WS		F = 0.15, *p* = 0.70, PES = 0.005		GR		F = 0.12, *p* = 73, PES = 0.004	
	D6				D3				Level				U3				U6		
	MEAN		STD		MEAN		STD		MEAN		STD		MEAN		STD		MEAN		STD
K	6.92		2.69		6.07		2.38		4.07		1.64		4.97		1.84		5.79		2.11
C	6.21		1.45		5.13		1.05		5.58		1.35		5.21		1.34		4.48		1.04
K *vs.* C	D6		(L,U)		D3		(L,U)		Level		(L,U)		U3		(L,U)		U6		(L,U)
	0.43		(−1.01, 2.32)		*p* = 0.2		(−0.51, 2.32)		*p* = 0.01		(−2.69, −0.37)		*p* = 0.65		(−1.52, 0.97)		*p* = 0.06		(−0.01, 2.55)
	D6 *vs.* D3		D6 *vs.* Level		D6 *vs.* U3		D6 *vs.* U6		D3 *vs.* Level		D3. *vs.* U3		D3 *vs.* U6		Level *vs.* U3		Level *vs.* U6		U3 *vs.* U6
K	*p* = 0.02		*p* = ∗∗∗		*p* = ∗∗∗		*p* = 0.02		*p* = ∗∗∗		*p* = 0.02		*p* = ∼ 1		*p* = 0.09		*p* = ∗∗∗		*p* = 0.14
C	*p* = ∗∗		*p* = ∼ 1		*p* = 0.1		*p* = ∗∗∗		*p* = ∼ 1		*p* = ∼ 1		*p* = 0.29		*p* = ∼ 1		*p* = 0.02		*p* = 0.27
StepWV																			
IN		F = 4.74, *p* = ∗∗, PES = 0.149		IN X WS		F = 0.5, *p* = 0.72, PES = 0.018		IN X GR		F = 1.96, *p* = 0.11, PES = 0.068		WS		F = 0.88, *p* = 0.36, PES = 0.031		GR		F = 0.89, *p* = 0.35, PES = 0.032	
	D6				D3				Level				U3				U6		
	MEAN		STD		MEAN		STD		MEAN		STD		MEAN		STD		MEAN		STD
K	15.56		4.57		12.9		4.05		9.79		1.66		10.97		3.58		13.14		4.5
C	15.51		2.79		12.67		1.33		11.15		2.96		12.85		2.86		14.67		4.02
StepTV																			
IN		F = 6.13, *p* = ∗∗, PES = 0.185		IN X WS		F = 0.78, *p* = 0.49, PES = 0.028		IN X GR		F = 13.56, *p* = ∗∗∗, PES = 0.334		WS		F = 0.42, *p* = 0.53, PES = 0.015		GR		F = 0.001, *p* = 0.98, PES = ∼0	
	D6				D3				Level				U3				U6		
	MEAN		STD		MEAN		STD		MEAN		STD		MEAN		STD		MEAN		STD
K	5.35		2.16		4.66		1.65		3.46		1.44		3.86		1.54		4.64		2
C	5.44		1.62		4.64		1.19		4.18		1.09		3.99		1.15		3.38		1.16
K *vs.* C	D6		(L,U)		D3		(L,U)		Level		(L,U)		U3		(L,U)		U6		(L,U)
	*p* = 0.82		(−1.62, 1.30)		*p* = 0.89		(−1.16, 1.02)		*p* = 0.13		(−1.73, 0.23)		*p* = 0.7		(−1.24, 0.84)		*p* = 0.052		(−0.01, 2.51)
	D6 *vs.* D3		D6 *vs.* Level		D6 *vs.* U3		D6 *vs.* U6		D3 *vs.* Level		D3. *vs.* U3		D3 *vs.* U6		Level *vs.* U3		Level *vs.* U6		U3 *vs.* U6
K	*p* = 0.03		*p* = ∗∗∗		*p* = ∗∗∗		*p* = 0.15		*p* = ∗∗∗		*p* = ∗∗∗		*p* = ∼ 1		*p* = ∗∗∗		*p* = ∗∗∗		*p* = ∗∗∗
C	*p* = 0.01		*p* = ∗∗		*p* = ∗∗∗		*p* = ∗∗∗		*p* = 0.03		*p* = ∗∗∗		*p* = ∗∗∗		p = 0.19		*p* = ∗∗∗		*p* = ∗∗
StrideTV																			
IN		F = 7.43, *p* = ∗∗∗, PES = 0.217		IN X WS		F = 1.49, *p* = 0.21, PES = 0.052		IN X GR		F = 9.51, *p* = ∗∗∗, PES = 0.261		WS		F = ∼0, *p* = 0.99, PES = ∼0		GR		F = 0.88, *p* = 0.35, PES = 0.032	
	D6				D3				Level				U3				U6		
	MEAN		STD		MEAN		STD		MEAN		STD		MEAN		STD		MEAN		STD
K	3.61		1.32		3.01		1.03		2.21		0.79		2.57		0.95		3.15		1.27
C	3.66		0.88		2.75		0.75		2.48		0.34		2.28		0.65		1.98		0.51
K *vs.* C	D6		(L,U)		D3		(L,U)		Level		(L,U)		U3		(L,U)		U6		(L,U)
	*p* = 0.81		(−0.97, 0.76)		*p* = 0.48		(−0.45, 0.94)		*p* = 0.32		(−0.69, 0.23)		*p* = 0.32		(−0.32, 0.94)		*p* = ∗∗		(0.43, 1.92)
	D6 *vs.* D3		D6 *vs.* Level		D6 *vs.* U3		D6 *vs.* U6		D3 *vs.* Level		D3. *vs.* U3		D3 *vs.* U6		Level *vs.* U3		Level *vs.* U6		U3 *vs.* U6
K	*p* = 0.02		*p* = ∗∗∗		*p* = ∗∗∗		*p* = 0.37		*p* = ∗∗		*p* = 0.02		*p* = ∼ 1		*p* = 0.28		*p* = ∗∗∗		*p* = ∗∗
C	*p* = ∗∗∗		*p* = ∗∗∗		*p* = ∗∗∗		*p* = ∗∗∗		*p* = ∼ 1		*p* = ∗∗		*p* = ∗∗		*p* = ∼ 1		*p* = 0.23		*p* = 0.73

### The effect of inclines

A significant main effect of incline was observed for step length (*F* = 19.36, *p* < 0.001), stride length (*F* = 28.44, *p* < 0.001), step time (*F* = 10.69, *p* < 0.001), and stride time (*F* = 11.23, *p* < 0.001). Specifically, step/stride length and step/stride time increased in both groups as the incline progressed from −6% to +6%.

### Linear and quadratic trend analysis of spatiotemporal gait variability

Linear Mixed Models (Test of Fixed effect) indicated significant quadratic trends in double support time (*p* = 0.019) and double support time variability (*p* = 0.004). The trend in double support time variability was particularly strong in bilateral KOA patients (*r* = 0.464, *p* < 0.001). Significant quadratic trends were also observed for step length variability (*p* = 0.05) and stride length variability (*p* = 0.05), again primarily in bilateral KOA patients (Step: *r* = 0.45, *p* < 0.001; Stride: *r* = 0.38, *p* = 0.001). Finally, stride time variability showed a significant trend (*p* = 0.033) in both bilateral KOA patients (*r* = 0.401, *p* < 0.001) and healthy controls (*R* = 0.672, *p* < 0.001). More details are shown in Table 4.

**Table 4:** Trend analysis using Linear Mixed Model for each dependent variable. DST, double support time; DSTV, double support time variability; ST, stance time; STV, stance time variability; StepL, step length; StepLV, step length variability; StrideL, stride length; StrideLV, stride length variability; StepWidth, step width; StepWidthV, step width variability; StrideT, stride time; StrideTV, stride time variability; StepT, step time; StepTV, step time variability; KOA, bilateral KOA patients.

DST	*p* value		DSTV	*p* value	
Linear X Group	*p* = 0.906		Linear X Group	*p* = 0.736	
Quadratic X Group	*p* = 0.019		Quadratic X Group	*p* = 0.004	
**KOA**	***p* value**	**R value**	**KOA**	***p* value**	**R value**
Linear	0.706	NA	Linear	*p* = 0.444	NA
Quadratic	0.076	NA	Quadratic	*p* < 0.001	*R* = 0.464, *p* < 0.001
**Controls**	***p* value**	**R value**	**Controls**	***p* value**	**R value**
Linear	0.657	NA	Linear	*p* = 0.064	NA
Quadratic	0.087	NA	Quadratic	*p* = 0.396	NA
**ST**	***p* value**		**STV**	***p* value**	
Linear X Group	*p* = 0.718		Linear X Group	*p* = 0.922	
Quadratic X Group	*p* = 0.048		Quadratic X Group	*p* = 0.629	
**KOA**	***p* value**	**R value**	**KOA**	***p* value**	**R value**
Linear	0.866	NA	Linear	NA	NA
Quadratic	0.132	NA	Quadratic	NA	NA
**Controls**	***p* value**	**R value**	**Controls**	***p* value**	**R value**
Linear	0.435	NA	Linear	NA	NA
Quadratic	0.216	NA	Quadratic	NA	NA
**StepL**	***p* value**		**StepLV**	***p* value**	
Linear X Group	*p* = 0.682		Linear X Group	*p* = 0.880	
Quadratic X Group	*p* = 0.642		Quadratic X Group	*p* = 0.05	
**KOA**	***p* value**	**R value**	**KOA**	***p* value**	**R value**
Linear	NA	NA	Linear	*p* = 0.396	NA
Quadratic	NA	NA	Quadratic	*p* = 0.003	*R* = 0.45, *p* < 0.001
**Controls**	***p* value**	**R value**	**Controls**	***p* value**	**R value**
Linear	NA	NA	Linear	*p* = 0.247	NA
Quadratic	NA	NA	Quadratic	*p* = 0.006	NA
**StrideL**	***p* value**		**StrideLV**	***p* value**	
Linear X Group	*p* = 0.839		Linear X Group	*p* = 0.880	
Quadratic X Group	*p* = 0.851		Quadratic X Group	*p* = 0.05	
**KOA**	***p* value**	**R value**	**KOA**	***p* value**	**R value**
Linear	NA	NA	Linear	*p* = 0.396	NA
Quadratic	NA	NA	Quadratic	*p* = 0.002	*R* = 0.38, *p* = 0.001
**Controls**	***p* value**	**R value**	**Controls**	***p* value**	**R value**
Linear	NA	NA	Linear	*p* = 0.171	NA
Quadratic	NA	NA	Quadratic	*p* = 0.429	NA
**StepWidth**	***p* value**		**StepWidthV**	***p* value**	
Linear X Group	*p* = 0.848		Linear X Group	*p* = 0.396	
Quadratic X Group	*p* = 0.147		Quadratic X Group	*p* = 0.238	
**KOA**	***p* value**	**R value**	**KOA**	***p* value**	**R value**
Linear	NA	NA	Linear	*p* = 0.396	NA
Quadratic	NA	NA	Quadratic	*p* = 0.238	NA
**Controls**	***p* value**	**R value**	**Controls**	***p* value**	**R value**
Linear	NA	NA	Linear	NA	NA
Quadratic	NA	NA	Quadratic	NA	NA
**StepT**	***p* value**		**StepTV**	***p* value**	
Linear X Group	*p* = 0.837		Linear X Group	*p* = 0.683	
Quadratic X Group	*p* = 0.969		Quadratic X Group	*p* = 0.059	
**KOA**	***p* value**	**R value**	**KOA**	***p* value**	**R value**
Linear	NA	NA	Linear	NA	NA
Quadratic	NA	NA	Quadratic	NA	NA
**Controls**	***p* value**	**R value**	**Controls**	***p* value**	**R value**
Linear	NA	NA	Linear	NA	NA
Quadratic	NA	NA	Quadratic	NA	NA
**StrideT**	***p* value**		**StrideTV**	***p* value**	
Linear X Group	*p* = 0.840		Linear X Group	*p* = 0.868	
Quadratic X Group	*p* = 0.946		Quadratic X Group	*p* = 0.033	
**KOA**	***p* value**	**R value**	**KOA**	***p* value**	**R value**
Linear	NA	NA	Linear	*p* = 0.342	NA
Quadratic	NA	NA	Quadratic	*p* = 0.001	*R* = 0.401, *p* < 0.001
**Controls**	***p* value**	**R value**	**Controls**	***p* value**	**R value**
Linear	NA	NA	Linear	*p* = 0.287	NA
Quadratic	NA	NA	Quadratic	*p* = 0.033	*R* = 0.672, *p* < 0.001

### Correlations between means of spatiotemporal parameters and pain, stiffness, and physical function

**Figure 4:**
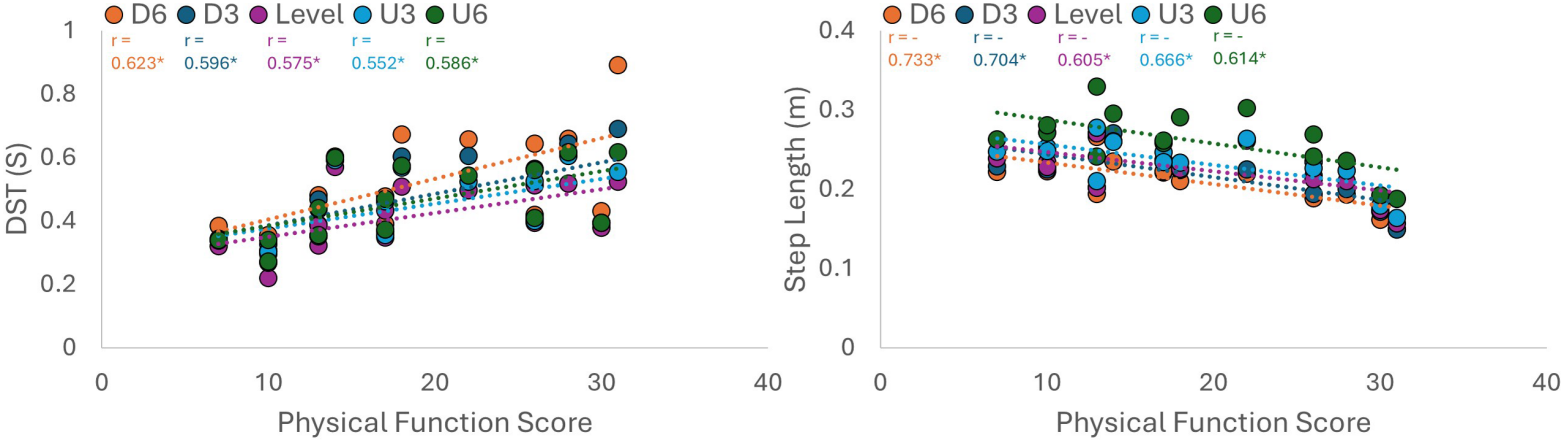
Correlation between WOMAC physical function score and gait parameters. Scatter plots illustrate the relationship between self-reported disability and objective gait mechanics. *: *p* < 0.05.

Pearson correlation analysis revealed significant, moderate correlations between the WOMAC physical function score and spatiotemporal parameters. Specifically, the relationship was positive for double support and stance time, but negative for step and stride length. No significant correlations were found between spatiotemporal parameters and the WOMAC pain or stiffness subscales. More details are shown in Table 5 and Fig. 4.

## Discussion

The altered spatiotemporal gait patterns associated with KOA have been well reported (Senden et al., 2011; Bolink et al., 2012; McCarthy et al., 2013; Rahman et al., 2015; Kluge et al., 2018; Hafer et al., 2020; Odonkor et al., 2020; Ismailidis et al., 2021; Boekesteijn et al., 2022). However, KOA gait alterations reported in previous studies are likely primarily due to reduced walking speed. Clinically, it is important to know if these spatiotemporal differences are still present between bilateral KOA patients and healthy controls when speed is controlled as a covariate during gait, as well as what adaptations occur in bilateral KOA patients while walking on level or inclined surfaces, as this is a common and necessary task in day-to-day living for complete rehabilitation. The results of the present study indicate that: (1) when walking speed was controlled as a covariate, significant differences persisted in double support time (specifically during downhill walking) and step/stride length (during both downhill and uphill walking), while stance time and step width did not differ significantly; and (2) patients with bilateral KOA exhibited a quadratic trend (U-shape) in gait variability across the five inclines (−6% to +6%) compared to the linear trend observed in controls, suggesting a distinct step-by-step control mechanism.

**Table 5:** The correlation between each dependent variable and Pain, Stiffness, and Physical function of WOMAC. DST, double support time; ST, stance time; StepL, step length; StrideL, stride length; StepWidth, step width; StrideT, stride time; StepT, step time. D6, −6% grade of incline; D3, −3% grade of incline; Level, 0% grade of incline; U3, 3% grade of incline; U6, 6% grade of incline. *: *p* < 0.05, **: *p* < 0.01.

		DST	ST	StepL	StrideL	StepWidth	StepT	StrideT
**PAIN**								
D6	Pearson Correlation	0.392	0.462	0.447	0.276	−0.122	0.356	0.363
	Sig. (2-tailed)	0.148	0.083	0.079	0.319	0.664	0.193	0.183
D3	Pearson Correlation	0.429	0.45	0.367	0.214	−0.376	0.316	0.307
	Sig. (2-tailed)	0.111	0.093	0.179	0.444	0.167	0.252	0.266
Level	Pearson Correlation	0.4	0.385	0.445	0.234	−0.248	0.372	0.372
	Sig. (2-tailed)	0.139	0.157	0.096	0.401	0.374	0.172	0.172
U3	Pearson Correlation	0.407	0.338	0.491	0.222	−0.307	0.415	0.392
	Sig. (2-tailed)	0.132	0.218	0.057	0.427	0.266	0.124	0.149
U6	Pearson Correlation	0.401	0.3	0.411	0.278	−0.129	0.35	0.368
	Sig. (2-tailed)	0.139	0.277	0.128	0.316	0.647	0.201	0.177
**STIFFNESS**									
D6	Pearson Correlation	0.368	0.452	−0.079	0.048	0.134	0.228	0.218
	Sig. (2-tailed)	0.177	0.09	0.78	0.864	0.633	0.414	0.435
D3	Pearson Correlation	0.215	0.352	−0.197	−0.006	−0.257	0.185	0.187
	Sig. (2-tailed)	0.441	0.198	0.482	0.983	0.355	0.509	0.505
Level	Pearson Correlation	0.126	0.222	−0.086	0.01	0.252	0.259	0.265
	Sig. (2-tailed)	0.655	0.426	0.759	0.973	0.365	0.351	0.341
U3	Pearson Correlation	0.069	0.189	−0.023	0.024	0.082	0.298	0.29
	Sig. (2-tailed)	0.806	0.499	0.935	0.932	0.771	0.281	0.294
U6	Pearson Correlation	0.134	0.058	−0.098	0.029	−0.075	0.372	0.384
	Sig. (2-tailed)	0.633	0.839	0.729	0.917	0.791	0.172	0.157
**PHYSICAL**								
D6	Pearson Correlation	.623^∗^	.605^∗^	−.733^∗∗^	−0.283	0.321	−0.449	0.376
	Sig. (2-tailed)	0.013	0.017	0.002	0.307	0.244	0.093	0.167
D3	Pearson Correlation	.596^∗^	.535^∗^	−.704^∗∗^	−0.249	0.269	−0.446	0.362
	Sig. (2-tailed)	0.019	0.04	0.003	0.37	0.333	0.096	0.185
Level	Pearson Correlation	.575^∗^	0.432	−.605^∗^	−0.201	0.149	−0.373	0.354
	Sig. (2-tailed)	0.025	0.108	0.017	0.473	0.596	0.17	0.195
U3	Pearson Correlation	.552^∗^	0.487	−.666^∗∗^	−0.239	0.471	−0.455	0.457
	Sig. (2-tailed)	0.033	0.065	0.007	0.392	0.076	0.088	0.087
U6	Pearson Correlation	.586^∗^	0.39	−.614^∗^	−0.311	.609^∗^	−0.43	0.405
	Sig. (2-tailed)	0.022	0.15	0.015	0.26	0.016	0.109	0.134

### Spatiotemporal adaptations and stability strategies

The results of this study demonstrated that navigating changing inclines required a coordinated adjustment of both spatial and temporal parameters in both populations. Surprisingly, despite functional limitations associated with bilateral KOA patients, these patients progressively increased their step and stride lengths as the incline increased from −6% to +6%. This trend may be explained by the distinct biomechanical constraints imposed by the different inclines. The shortest steps were observed during downhill conditions, likely because downhill walking demands significant eccentric muscle control to decelerate the body. As this eccentric capacity is often compromised in bilateral KOA patients and associated with joint instability (Suzuki et al., 2023), patients likely adopted a “braking strategy” with shortened steps to maintain control. Conversely, as the incline transitioned to uphill, the mechanical demand shifted toward concentric propulsion. Although uphill walking requires greater muscle activation (Sedaghatnezhad et al., 2021), it reduces impact transients and braking forces; therefore, patients may have felt sufficient stability to lengthen their stride relative to the highly constrained downhill condition.

However, despite this shared adaptive trend regarding slope, a distinct group difference persisted regardless of the incline condition. While healthy controls exhibited a gait pattern characterized by longer spatial parameters paired with shorter temporal durations, the bilateral KOA patients displayed a persistent, specific deficit in spatial output, manifesting as shorter step and stride lengths accompanied by longer step and stride times. This divergence suggests that bilateral KOA patients adopt a specific “stability-first” trade-off distinct from healthy mechanics. By sacrificing spatial efficiency through shorter step lengths, patients might prioritize the reduction of external kinetic forces; specifically, shorter steps have been shown to reduce both knee adduction moments (Ulrich et al., 2023) and knee flexion moments (Prentice et al., 2004). This spatial restriction effectively lowers the mechanical demand on the quadriceps and patellofemoral joint, serving as a protective mechanism against pain and joint loading (Ulrich et al., 2023). While this compensatory mechanism improves immediate stability, it comes at the cost of reduced walking efficiency, highlighting the complex functional adaptation required to navigate inclined surfaces in these bilateral KOA patients (Wang, Chien & He, 2025; Zhang et al., 2024).

In the current study, bilateral KOA patients exhibited significantly longer double support time (the phase when both legs are on the ground) compared to controls during both uphill and downhill walking. In contrast, healthy controls showed no significant variation in double support time across inclines relative to level walking, nor was a significant difference observed between the two groups during level walking. This latter finding contrasts with previous research, which reported higher double support time in bilateral KOA patients compared to healthy controls on level ground (Zhang et al., 2024). This discrepancy is likely explained by the methodological design of the current study, which controlled for walking speed as a covariate, as spatiotemporal differences often dissipate when groups are matched for walking speed. Bilateral KOA patients significantly increased their double support time on inclines while controls did not, despite speed matching, highlights the unique stability challenge imposed by inclined surfaces for this population. It is also possible that the K/L grades used for recruitment in their study (3–4) were higher than those in the present study (2). Although this adaptation was evident in both incline conditions, downhill walking appeared to present specific risks for bilateral KOA patients, evidenced by longer double support times compared to uphill walking (0.52 s for −6% grade *vs.* 0.46 s for 6% grade, *p* < 0.01). When walking downhill, gravity accelerates the body forward. In a healthy person, the leading leg catches the weight and uses the quadriceps to ‘brake’ immediately; however, in bilateral KOA patients, this braking mechanism is often compromised. Thus, we speculated that: (1) by keeping both feet on the ground longer, patients distribute the high eccentric braking forces across two limbs, preventing the weak quadriceps of the leading leg from being overwhelmed; and (2) this extended double support time constrains the center of mass within a stable base of support, providing a critical temporal buffer that allows the patient’s delayed proprioceptive system to verify joint stability before committing to the next step. Given that the mean WOMAC score in this study was 25.4, indicating a moderate impact on quality of life, the observed increase in double support time on inclines should be interpreted as a necessary compensatory strategy to enhance stability. Clinicians should therefore monitor patient response closely when introducing inclined gradients.

### Gait variability and motor control

Gait variability, defined as the step-to-step fluctuations in spatiotemporal parameters, is widely recognized as a predictor of fall risk, particularly in older adults and bilateral KOA patients. Increased variability in stride time, stride length, and double support time often reflects compensatory mechanisms utilized to maintain stability amidst pain, stiffness, and muscle weakness. In the current study, a significant interaction between group and incline was observed for these variability metrics. Specifically, bilateral KOA patients exhibited increased variability during both uphill and downhill walking compared to level ground, following a significant quadratic (U-shaped) trend (Figs. 2–3). In contrast, controls demonstrated a linear decrease in variability as the incline increased. Although traditional models view elevated variability strictly as a sign of instability, this finding can be better understood through the lens of Stergiou, Harbourne & Cavanaugh (2006)’s “optimal movement variability” framework, which posits that variability is a necessary component of adaptive motor control. From this perspective, the U-shaped elevation in variability represents the motor system actively searching for stable solutions to meet the heightened mechanical demands of the incline. Empirical evidence supports this premise; healthy young adults have been shown to exhibit similar U-shaped trends when navigating steeper inclines (−15% to +15%). The linear trend observed in our control group likely indicates that the current incline range (−6% to +6%) was insufficient to trigger this non-linear response in healthy individuals. Consequently, we propose that the U-shaped trend in gait variabilities observed in the bilateral KOA patients does not necessarily imply instability, particularly given the absence of falls or tripping incidents during these trials. Instead, as illustrated in the trendlines (Figs. 2–3), this modulation likely reflects a flexible, adaptive tuning of the motor system required to manage the distinct biomechanical constraints of inclined walking.

Another interesting observation was that the shape of the trend line (U-shape) was similar between stride and step length variabilities in bilateral KOA patients. This observation contrasts with results from split-belt gait adaptation paradigms (Gregory, Sup 4th & Choi, 2021), which revealed that the primary control mechanism was step length, which was based on foot placement (spatial parameters) but not stride length. The primary control mechanics for stride length were actually mixed spatiotemporal (step timing plus step length). Additionally, these previous studies showed that for after-effect persistence in split-belt treadmill adaptation, the step length symmetry was short-lived (diminished after 5 min rest); however, the stride length symmetry was partially retained after 5 min rest. This suggests that spatial (step length) and temporal (stride length) gait control exhibit distinct adaptation patterns during split-belt treadmill training, with implications for rehabilitation strategies, not similar to the findings of this study. In the current study, when walking on challenging locomotor behaviors such as uphill and downhill, the control mechanism of step length and stride length were similar by increasing the variabilities when angle of inclines changed. Therefore, these observations suggested that different types of locomotor tasks induced different control mechanisms in terms of step and stride length variabilities.

### Clinical associations

The lack of a significant association between spatial–temporal parameters and WOMAC Pain and Stiffness, as opposed to the Physical Function, subscales was likely to be the result of the different constructs these domains measure (Maly, Costigan & Olney, 2006; Stratford & Kennedy, 2006). The items included in the Physical Function subscale were related to difficulties experienced during dynamic tasks such as walking and stair-climbing, which can be directly linked to the biomechanics of treadmill walking used in the present study (Bellamy et al., 1988). This connection was therefore reflected in impairments in gait kinematics, namely, reduced stride length and increased double support time as specific functional limitations described by the subject. In contrast, the WOMAC Pain and Stiffness subscales include items related to static states (*e.g.*, night pain, pain on standing from a seated position), that may not influence immediate gait kinematics (Zeni & Higginson, 2011). These disconnects suggested that spatiotemporal changes in bilateral KOA patients more closely represented the functional mobility status of an individual rather than their subjective experience of pain, supporting the use of gait analysis as an objective measure of functional impairment (Ornetti et al., 2010). Also, downhill walking, particularly at −6%, appears to be the most sensitive condition for identifying objective functional limitations, as evidenced by its strong associations with reduced step length (*r* = −0.733, *p* = 0.002) and increased double support time (*r* = 0.623, *p* = 0.013). In contrast, while incline walking at +3% grade maintains a high correlation with step length (*r* = −0.666, *p* = 0.007), the steepest incline at +6% grade uniquely triggers a significant correlation with Step width (*r* = 0.609, *p* = 0.016), suggesting that individuals with greater physical impairment may adopt a wider base of support to maintain stability during steep ascents.

**Figure 5:**
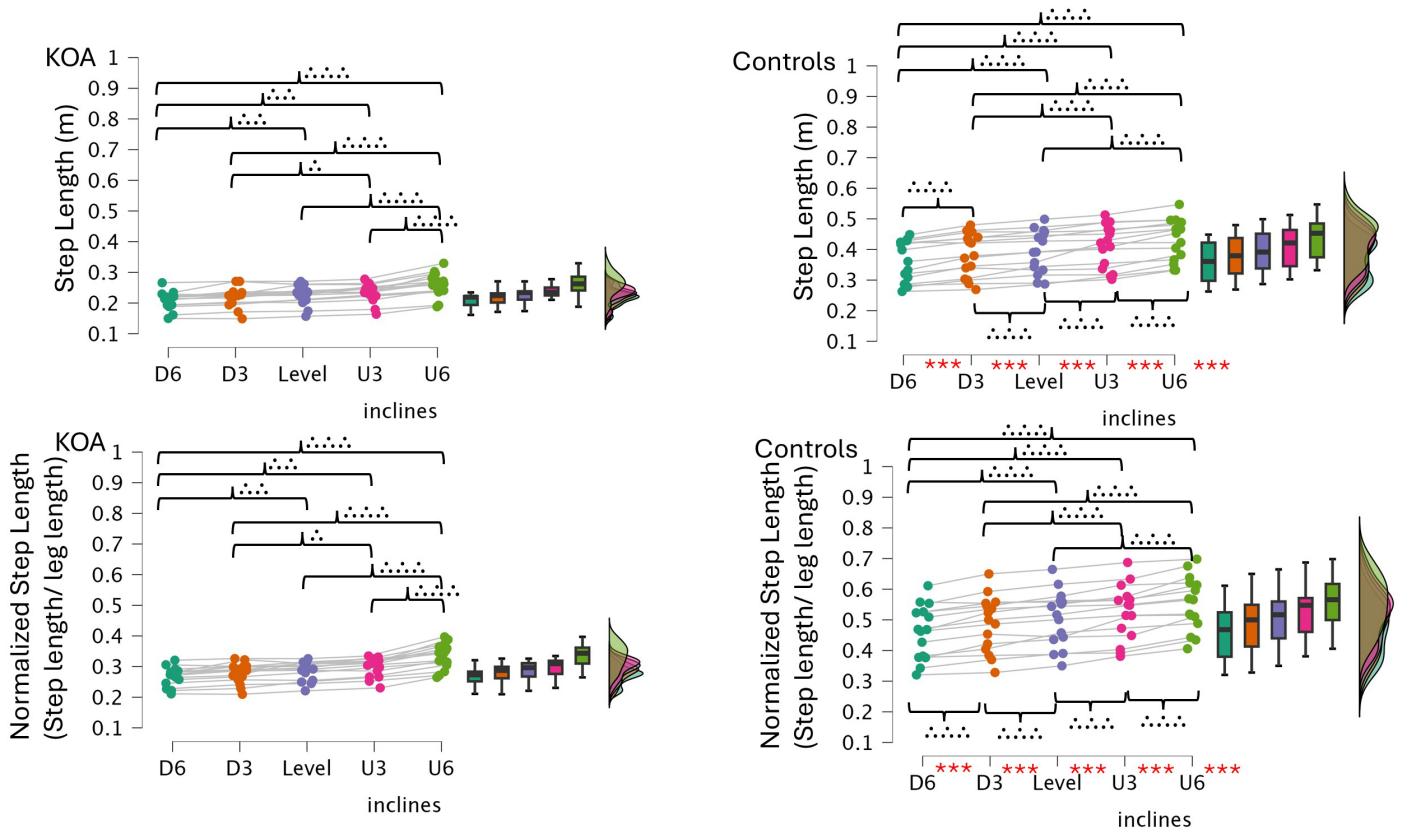
Step length and normalized step length across inclines in controls and bilateral KOA patients. These raincloud plots illustrate the distribution and individual data points for step length (m) and normalized step length (step length divided by leg length). Statistical significance for between-group comparisons is indicated by red asterisks, and within-group comparisons across inclines are indicated by brackets with dots (representing *: *p* < 0.05, **: *p* < 0.01, ***: *p* < 0.001).

**Figure 6:**
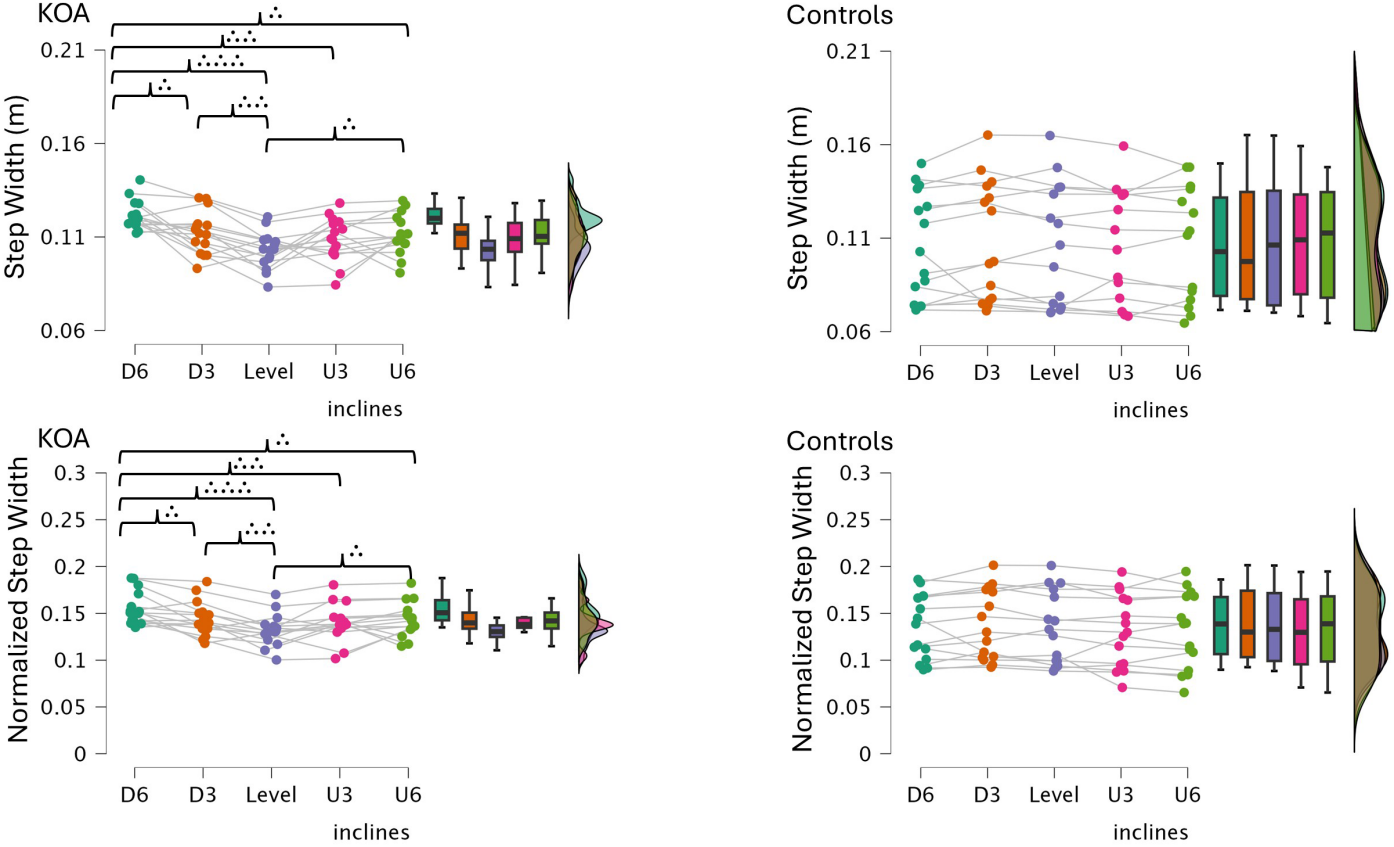
Step width and normalized step width across inclines in controls and bilateral KOA patients. These raincloud plots illustrate the distribution and individual data points for Step Width (m) and Normalized Step Width (step width divided by leg length). Statistical significance for between-group comparisons is indicated by red asterisks, and within-group comparisons across inclines are indicated by brackets with dots (representing *: *p* < 0.05, **: *p* < 0.01, ***: *p* < 0.001).

In gait analysis, normalizing spatial parameters to anatomical dimensions, such as leg length, is often preferred. This technique ensures that differences in step length reflect actual neuromuscular control rather than simply physical stature (Pinzone, Schwartz & Baker, 2016). However, in the current study, comparing absolute step length against normalized values produced nearly identical statistical results across all inclined conditions Figs. 5–6). Both methods revealed the same incline effects and group differences. This similarity is explained by our study design, which matched participants by body size. As shown in Table 1, the bilateral KOA patients and control groups had similar heights and leg lengths, effectively removing stature as a confounding variable. Consequently, mathematical normalization was not required to interpret the data, confirming that the observed gait changes resulted from the pathology and slope mechanics, rather than differences in body size.

## Conclusions

This study characterizes the distinct spatiotemporal gait adaptations of bilateral KOA patients during incline walking. Even when walking speed was controlled as a covariate, bilateral KOA patients exhibited persistent deficits, including significantly shorter step and stride lengths and longer double support times compared to healthy controls, adaptations that were most pronounced during downhill walking. A second notable difference in motor control emerged with gait variability: As incline increased, variability linearly decreased for healthy controls, whereas a quadratic, U-shaped trend was observed for bilateral KOA patients, suggesting a flexible, adaptive tuning of the motor system to accommodate stability demands at both uphill and downhill extremes. Together, these results indicate that a stability-first braking strategy is utilized by bilateral KOA patients to prioritize immediate safety and load distribution over efficiency in movement. Overall, these findings emphasize the clinical need for rehabilitation to specifically target the unique challenges to stability and eccentric braking demands associated with downhill walking for improved functional safety in daily living.

## Limitations

The primary limitation of this study was the small sample size; however, the effect sizes of significant variables were substantial (partial eta squared values: 0.42 for double support time and 0.18 for step width), indicating a large effect size. A second limitation concerns the calculation of step and stride lengths. While these parameters were adjusted using the cosine of the incline angle, the calculation did not explicitly correct for the minor discrepancy between treadmill belt speed and horizontal velocity. However, this difference is mathematically negligible. For example, at the steepest incline (+6%, approx. 3.43°), the cosine value is 0.998. For a participant walking at the maximum observed speed of 1.08 m/s, the corresponding horizontal velocity would be 1.078 m/s. This represents a deviation of approximately 0.002 m/s, which may be ignored. Third, the study population was restricted to bilateral KOA patients to specifically examine systemic adaptations rather than inter-limb compensation. Future research comparing these results with unilateral KOA populations would be beneficial to better isolate strategies of inter-limb compensation. Finally, the analysis was restricted to a standardized window of the first 40 gait cycles (80 steps) for all participants to match the capacity of bilateral KOA patients under challenging incline conditions. While larger stride counts are sometimes recommended, recent evidence suggests that as few as 15 strides provide reliable and valid recordings of gait variability in clinical populations (Kroneberg et al., 2019). By utilizing a 40-cycle window, this study exceeded these minimum reliability thresholds while ensuring that data were not confounded by the onset of physical fatigue.

##  Supplemental Information

10.7717/peerj.20910/supp-1 Supplemental Information 1 Data

## Additional Information and Declarations

### Data Availability

The following information was supplied regarding data availability: The raw data are available in the Supplemental File.
